# Specialized pro-resolving lipid mediators regulate inflammatory macrophages: A paradigm shift from antibiotics to immunotherapy for mitigating COVID-19 pandemic

**DOI:** 10.3389/fmolb.2023.1104577

**Published:** 2023-02-03

**Authors:** Vikram Kumar, Nusrath Yasmeen, Anis Ahmad Chaudhary, Abdullah S. Alawam, Mohammed Al-Zharani, Nosiba Suliman Basher, S. Harikrishnan, Muddapuram Deeksha Goud, Aishwarya Pandey, Sudarshan Singh Lakhawat, Pushpender Kumar Sharma

**Affiliations:** ^1^ Amity institute of Biotechnology, Amity University Rajasthan, Jaipur, Rajasthan, India; ^2^ Department of Biology, College of Science, Imam Mohammad Ibn Saud Islamic University (IMSIU), Riyadh, Saudi Arabia; ^3^ INRS, Eau Terre Environnement Research Centre, Québec, QC, Canada

**Keywords:** COVID-19, antimicrobial, cytokine storm, macrophages, lipid mediators

## Abstract

The most severe clinical manifestations of the horrifying COVID-19 disease, that claimed millions of lives during the pandemic time, were Acute respiratory distress syndrome (ARDS), Coagulopathies, septic shock leading eventually to death. ARDS was a consequence of Cytokine storm. The viral SARS-COV2infection lead to avalanche of cytokines and eicosanoids causing “cytokine storm” and “eicosanoid storm.” Cytokine storm is one of the macrophage-derived inflammatory responses triggered by binding of virus particles to ACE2 receptors of alveolar macrophages, arise mainly due to over production of various pro-inflammatory mediators like cytokines, e.g., interleukin (IL)-1, IL-2, and tumor necrosis factor (TNF)- α, causing pulmonary edema, acute respiratory distress, and multi-organ failure. Cytokine storm was regarded as the predictor of severity of the disease and was deemed one of the causes of the high mortality rates due to the COVID-19. The basis of cytokine storm is imbalanced switching between an inflammation increasing - pro-inflammatory (M1) and an inflammation regulating-anti-inflammatory (M2) forms of alveolar macrophages which further deteriorates if opportunistic secondary bacterial infections prevail in the lungs. Lack of sufficient knowledge regarding the virus and its influence on co-morbidities, clinical treatment of the diseases included exorbitant use of antibiotics to mitigate secondary bacterial infections, which led to the unwarranted development of multidrug resistance (MDR) among the population across the globe. Antimicrobial resistance (AMR) needs to be addressed from various perspectives as it may deprive future generations of the basic health immunity. Specialized pro-resolving mediators (SPMs) are generated from the stereoselective enzymatic conversions of essential fatty acids that serve as immune resolvents in controlling acute inflammatory responses. SPMs facilitate the clearance of injured tissue and cell debris, the removal of pathogens, and augment the concentration of anti-inflammatory lipid mediators. The SPMs, e.g., lipoxins, protectins, and resolvins have been implicated in exerting inhibitory influence on with cytokine storm. Experimental evidence suggests that SPMS lower antibiotic requirement. Therefore, in this review potential roles of SPMs in enhancing macrophage polarization, triggering immunological functions, hastening inflammation resolution, subsiding cytokine storm and decreasing antibiotic requirement that can reduce AMR load are discussed.

## Graphical Abstract

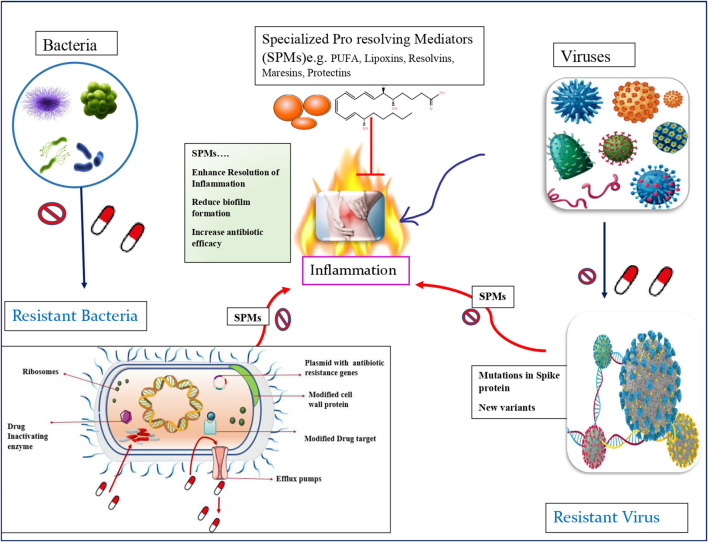

## Introduction

At the time of publication of this article a total of 113 million individuals have been clinically affected by the current pandemic. Of these, approximately 89 million have recovered, 21 million still have not and more than 2 million lives have been lost to the disease. The causative organism for the coronavirus disease 2019 (COVID-19) is the Severe Acute Respiratory Syndrome Coronavirus 2 (SARS-CoV-2), which is an enveloped, single-stranded, positive-sense RNA virus, that belongs to the group of Beta coronaviruses 2b lineage of the family coronaviridae, and the order Nidovirales (Huang et al., 2020; Hoffmann et al., 2020; Rastogi et al., 2020). It closely resembles SARS-CoV as 79.5% of its genomic sequence is identical to the latter (Shin et al., 2020; Zhou F et al., 2020; Zhou P et al., 2020). SARS-CoV-2 has spherical morphology, with spike-like glycoproteins (S proteins) protruding from its surface. Their S1 and S2 subdomains are crucial in host cell receptor binding and fusion with cell membrane. The former shows more variation across the receptor-binding domain (RBD), whereas the S2 subdomain is conserved, comprising of the fusion machinery necessary for the virus to reach the host cell (Shin et al., 2020).

Co-infections and superinfections that occur in conjunction with viral respiratory infections can significantly increase the mortality rate of patients, as pointed out in laboratory, clinical, and epidemiological studies (Metzger and Sun, 2013; Paget and Trottein, 2019). Bacterial co-infections were specifically recorded as being able to alter the mortality rate in viral infections, of which influenza-related bacterial infections are a prime example (Smith and McCullers, 2014; Jia et al., 2017; Katsurada et al., 2017; Quah et al., 2018). *Streptococcus* pyogenes, *Neisseria* meningitidis, *Moraxella catarrhalis*, *Streptococcus pneumoniae*, *Haemophilus* influenzae, and *Staphylococcus aureus* are all capable of causing influenza-related infections (Abbasi et al., 1994; Jacobs et al., 2014; Smith and McCullers, 2014; Mulcahy and McLoughlin, 2016; Ochi et al., 2018; Su et al., 2019). Antibiotics are being used in abundance despite COVID-19 being caused by a virus to treat or prevent secondary infections (Zhou, F et al., 2020; Chedid et al., 2021). This is cause for concern as there is a lack of new antibiotics to deal with drug-resistant bacterial infections, which arose due to the overuse of antibiotics in the first place. (Rawson et al., 2020). Additionally, the use of sanitizers and disinfectants, that has increased severalfold as measures to prevent infection spread increase the risk of antimicrobial resistance (Egyir et al., 2020).

COVID-19 progression involves pulmonary hyper-inflammation and release of pro-inflammatory cytokines (e.g., TNF-α, IL-6, IL-1, IL-8, and MCP-1), as part of “cytokine storms” (Mehta et al., 2020). Cell death and the resulting cell debris accumulation triggers inflammasomes, which bring about a surge of pro-inflammatory bioactive lipid mediators like prostaglandins and leukotrienes (“eicosanoid storm”) resulting in local inflammation (von Moltke et al., 2012). Management of these local and systematic responses are as crucial an antiviral therapy. Eicosanoids are endogenous lipid autacoid mediators capable of inducing inflammation, while endogenous pro-resolution lipids are anti-inflammatory agents that can terminate the inflammatory response by promoting the removal of cellular debris (Doolittle, 1998). Specialized pro-resolving mediators (SPMs) are bioactive lipid autacoids that can stimulate phagocytosis of cellular debris by macrophages as well as counter the release of pro-inflammatory cytokines/chemokines, thereby bringing about endogenous resolution of inflammatory response. Discovery of SPMs such as resolvins, lipoxins, and protectins has given traction to the idea that inflammation resolution is an active biochemical process (Serhan, 2014).

Alveolar macrophages may be integral to COVID-19 disease progression and disease-induced deaths. Infection of lungs by SARS-CoV-2 virus followed by failure to rapidly remove the virus leads to severe inflammatory response, tissue damage and fibrosis. Prolonged overactivation of macrophages and increased release of pro-inflammatory cytokines result in clinical manifestations that are markedly identical to those of macrophage activation syndrome (MAS). This being the case, identifying methods to regulate macrophage response in cases of severe COVID-19 is key to recovery (Tang L et al., 2020). In this review, we discuss the role of Specialized Pro-resolving lipid Mediators (SPMs) in driving the macrophages immuno-functions and thus regulating the macrophage-induced inflammation. Moreover, we discuss the clinical potentials of SPMs in COVID-19 treatment.

## Antimicrobial resistance in the COVID-19 ERA

Antimicrobial resistance (AMR) emerged as a notable issue quite a while before the start of the COVID-19 pandemic. With the influx of novel antibiotics at a decline since the 1980s, instances of reported cases of infections by drug-resistant organisms have soared (Christaki et al., 2020). COVID-19, being caused by the virus SARS-CoV-2, one might assume that antibiotics are of little to no use. However, there are a few factors that necessitate the use of antimicrobials. One of these reasons is that symptoms of COVID-19 closely resemble those of bacterial pneumonia and as such, antibiotics are prescribed immediately in severe cases prior to confirmation *via* diagnostic testing. Another reason is secondary infections associated with COVID-19, where antimicrobial use is required to kill invading bacteria and fungi (Knight et al., 2021). Besides prescription, individuals also consumed pharmaceuticals compounds whose efficacy was unproven under the influence of social media discourse (Afshinnekoo et al., 2021). These situations led to the use and abuse of antimicrobials during the pandemic, and it is precisely their use that leads to antimicrobial resistance. The CDC (Centers for Disease Control and Prevention, United States of America) released a 2022 special report on antimicrobial resistance, highlighting the changes in trends that occurred during the response to COVID-19. The report considered 18 antimicrobial resistance threats, and for these data was unavailable for 9, while all the rest showed significant increase in infection rates (CDC, 2022). Overall, cases of antimicrobial resistance have increased in the COVID-19 era.

### Choice of antibiotics for COVID-19 patients

The CDC reported that during the pandemic response, 80% of patients admitted to US hospitals were given antibiotic treatment, either due to their condition being confused with bacterial pneumonia or because of secondary infections. The two most common antibiotics prescribed were azithromycin and ceftriaxone (CDC, 2022). Clinicians were concerned with two priorities while prescribing antibiotics—one was prescribing an antibiotic with a broad enough spectrum to be effective against the organism, while the other involved not using last resort antibiotics and preventing wastage (Knight et al., 2021). Secondary infections with viral diseases are usually due to *Staphylococcus aureus*, *Streptococcus pneumoniae*, *Neisseria* meningitides, *Haemophilus* influenzae, *Klebsiella pneumoniae*, and members of the genus *Proteus*, *Enterobacter*, and *Citrobacter* species. These are present in the hospital setting, and their spread is nosocomial (Manohar et al., 2020). Drug-resistant organisms, such as multidrug-resistant *Escherichia coli*, *Enterococcus*, *Chlamydia pneumoniae*, *Klebsiella* pneumonia, *Pseudomonas aeruginosa*, *Mycoplasma* pneumonia, and extended-spectrum beta-lactamase are associated with SARS-CoV-2 infections (Adebisi et al., 2021). It was reported that the following antibiotics were used to treat COVID-19: cephalosporins, quinolones, carbapenems, tigecycline against methicillin-resistant *Staphylococcus aureus*, linezolid, antifungal drugs (Chen N et al., 2020). However, the usage of several other antibacterials was noted such as moxifloxacin, ceftriaxone, and azithromycin (Wang et al., 2020). The best way to prevent prescription and eventual abuse of antibiotics remain prevention of infections from occurring in the first place.

### Antimicrobial resistance pattern in COVID-19

Antimicrobial resistance emerges in response to changes—man-made or natural—that might occur in their environment. Generally, mutation of genes and horizontal transfer of existing resistance genes can result in resistance to antimicrobial agents (Boerlin & Reid-Smith, 2008). Going into the pandemic, warnings were issued that prolonged and inappropriate use of antimicrobials will exacerbate the problem of AMR, such that their spike in use will result in more deaths in the future (Miranda et al., 2020). The general mechanisms of antimicrobial resistance are antibiotic destruction or modification through enzymatic means, alteration to target of antibiotic action, changes to membrane permeability and pumping out antimicrobial compounds *via* efflux pumps (Christaki et al., 2020). The major change introduced during the pandemic was the frequency with which biocides and pharmaceutical compounds were brought in contact with microbes in the body and the environment. These involve alcohol-based hand sanitizers, surfactants, phenols, quaternary ammonium compounds, hydrogen peroxide and others (Lobie et al., 2021). Mutagenesis-induced tolerance of alcohol had been reported in bacteria even before the start of the pandemic (Pidot et al., 2018). Frequent use of antimicrobial agents places stress on organisms, resulting in selection of colonies capable of withstanding the effects of these compounds. Prolonged exposure can thus result in these compounds becoming ineffective (Christaki et al., 2020; Miranda et al., 2020).

## SPMs in infection

Specialized pro resolving mediators (SPMs) are endogenous lipid mediators, produced from innate immune cells, formed *via* stereoselective enzymatic conversion of essential fatty acids like arachidonic acid, eicosapentaenoic acid (EPA, C20:5n-3), n-3 docosapentanoic acid and docosahexaenoic acid (DHA, C22:6–3) (Lee, 2021). The different subgroups of SPMs identified are: resolvins (Rvs), protectins, maresins, and lipoxins. SPMS, are antimicrobial in nature, clear the debris of killed pathogens by immunocytes, facilitate the clearance of injured tissue and cell debris. They inhibit PMN infiltration and recruitment, enhance macrophage phagocytosis, and stimulate efferocytosis by activating macrophage polarization from M1 to M2 (Akagi et al., 2015; Kang and Lee., 2016). Furthermore, SPMs decrease the release of pro-inflammatory chemical mediators, whilst increase the anti-inflammatory mediators such as IL-10, thereby reduces both cytokine and eicosanoid storms (Schif-Zuck et al., 2011; Titos et al., 2016; Recchiuti et al., 2011). Finally, they exhibit anti-nociceptive effects by reducing inflammatory pain, stimulate tissue regeneration, and enhance wound healing (Allen et al., 2020; Serhan et al., 2012; Regidor et al., 2020). SPMs serve as immune resolvents in controlling acute inflammatory responses and augment the concentration of anti-inflammatory lipid mediators. Evidence from several studies, signify the role of SPMs in the modulation of host responses to various infectious diseases, and hence can be ventured as a new therapeutic opportunity for treating infectious inflammatory diseases, and to curtail the usage of antibiotic therapy, with prime focus to overcome antimicrobial resistance (Zhao et al., 2021; Chiang et al., 2019; Basil and Levy, 2016).

### SPMs in bacterial infection

Studies conducted on bacterial infection models revealed varied levels of SPMs. A non-human primate model of pulmonary infection showed notable decrease in levels of RvE1 in blood, when the infection was induced by Streptococcal pneumonia (Dalli et al., 2015a). Similarly, lowered RvD1 levels were present in a rodent model of *Pseudomonas aeruginosa* induced pneumonia (Codagnone et al., 2018). These incidences seem to suggest that a drop in the formation of SPMs is involved in pathogenicity of pneumonia. A compilation of results from several studies imply that increased production of SPMs is important in host defense for bacterial clearance and infection resolution (Dalli et al., 2015b; de la Rosa, X et al., 2018; Thornton and Yin, 2021). Conversely, inability to survive infections could be attributed to a failure to resolve the inflammatory response, owing to the decrease in SPM levels (Dalli et al., 2017). Though dietary precursors of SPMs are capable of raising their levels, use of pure SPMs may be required in cases of acute bacterial infections (Thornton and Yin, 2021). RvD2 and LxA4 are prime candidates for this purpose, as their effectiveness has been established in preclinical rodent models, and they have no correlation with sepsis non-survivors (Walker et al., 2011; Chiang et al., 2017; Dalli et al., 2017). Other studies have suggested the role of endogenously formed SPMs in the pathophysiology of bacterial infection. They also point to specific SPMs as biomarkers to assess disease severity (Basil and Levy, 2016). The majority of research into the utility of SPMs as therapeutic compounds have focused on their antibiotic mechanisms, optimal dose and timing of administration in order to maximize their effectiveness (Thornton and Yin, 2021). Using these compounds to combat bacterial infection might reduce the world’s dependence on antibiotics, and potentially alleviate the evolving global crisis of antimicrobial resistance (Basil and Levy, 2016; Thornton and Yin, 2021), as discussed in Table 1.

**TABLE 1:** SPMs influence host defense and infectious inflammation.

SPM	Role	Mediators	Receptors	Examples of infection
Bacterial infections	Reducing neutrophils migration and declining pro-inflammatory T cell activity (Thornton and Yin, 2021)	Lipoxins	RvE1	*Mycobacterium* Tuberculosis, *Salmonella* spp., Sepsis, *Escherichia coli*, *Staphylococcus aureus* and Streptococcal pneumonia
		Resolvins	RvD1	
			RvD2	
			LxA4	
Virus infections	Inhibits viral RNA nuclear export, rise antibody production by augmenting B-cell differentiation to an antibody secreting B-cell (Genis et al., 1992; Cilloniz et al., 2010; Rajasagi et al., 2011; Morita et al., 2013; Basil and Levy, 2016; Balta et al., 2021)	Lipoxins	LxA4	Influenza A, RSV, HIV, HSV
		Resolvins	D1	
		Protectins	D2	
Parasitic infections	Decrease of recruitment of neutrophils, lymphocytes and eosinophils (Aliberti et al., 2002)	Lipoxins	IL12	Toxoplasma gondii, Angiostrongylus costaricensis, Plasmodium spp. and Trypanosoma cruzi
			LxA4	
			IFNγ	

### SPMs in viral infections

Viruses, being obligate parasites, must infect target cells and highjack their cellular machinery to replicate. The stages involved in this process constitute the viral life cycle. These are: entry, genome replication, and exit (Ryu, 2017; Lee, 2021). The first of these stages can be subdivided into attachment, penetration and uncoating, while the last one includes virion assembly and release. SPMs are known to affect the inflammatory response generated in response to a viral infection and their basic roles, mediators and receptors are depicted in Table 1. However, studies that examine the direct effect of these molecules on viral life cycle are very few. There has been a recent report of LXA4 being able to influence the life cycle of Kaposi’s Sarcoma-Associated Herpesvirus (KSHV). This is achieved through chromatin modulation and hedgehog signaling, and the net result is that the dormancy of the virus is destabilized and the expression of programmed death-ligand 1 (PD-L1) is reduced in Kaposi’s sarcoma. As a result of these effects immune evasion is reduced (Asha et al., 2020). On the other hand, there are more than a few reports of SPM receptors acting as receptors for viruses as well. This intersection of host immune system with points in viral infection pathway presents new opportunities for utilizing SPMs. Provided that the duration of necessary immune response and the point at which the response must be suppressed can be determined, SPMs can be used to prevent damage to the host. More research is needed to determine these parameters and to employ these mediators in case of viral infection (Basil and Levy, 2016).

### SPMs in parasitic infections

SPMs do play a part in the immune response elicited by parasitic infections. An intense response involving dendritic cells (DCs) and production of IL-12 takes place during infection by Toxoplasma gondii (Reis e Sousa et al., 1997). Lipoxins produced during toxoplasmosis employ an autocoid mechanism on DCs *via* ALX, through which expression of CCR5 and IL-12 production are attenuated (Aliberti et al., 2002) as depicted in Table 1. Also, lowered levels of 5-LOX in animal models can result in higher amounts of IL-2 and IFNγ relative to those of wild-type animals, severe encephalitis and increased risk of death, which can all be relieved through the introduction of LXA4 analogues (Aliberti et al., 2002b). Administration of lipoxins is also reported to aid host defense in case of both intracellular and extracellular parasitic infection, including Angiostrongylus costaricensis (Bandeira-Melo et al., 2000), Plasmodium spp. (Shryock et al., 2013) and Trypanosoma cruzi (Molina-Berríos et al., 2013).

## SPMs in regulation of inflammatory macrophages in COVID-19

### Macrophages in COVID-19

**FIGURE 1:**
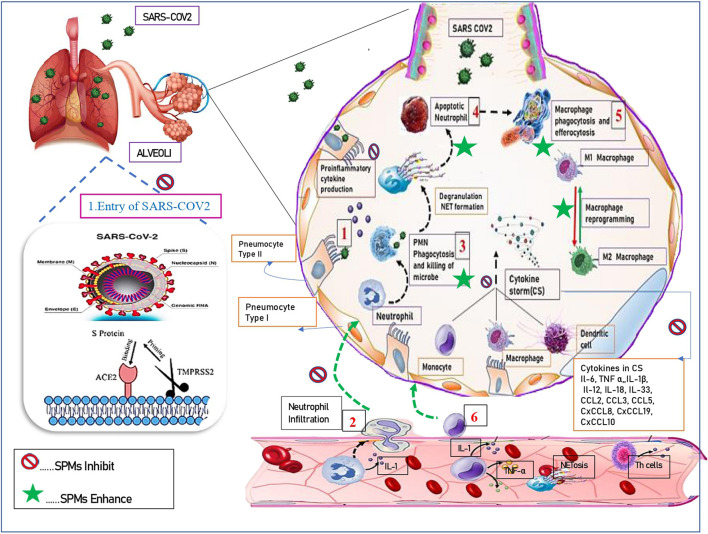
Resolution of inflammation using SPMs. 1) Resident cells detect the stimulus and initiate the inflammatory cascade. 2). There is infiltration of granulocytes [such as neutrophils, monocytes. 3) PMN Phagocytosis and killing of microbes. 4) Apoptosis of neutrophils after, degranulation and NET formation. 5) Macrophage phagocytosis and efferocytosis. 6) Monocytes/macrophages, from the blood into the affected tissue. Resolution of inflammation is enhanced by SPMs wherein they kill microorganisms, stop entry of virus. Inhibit secretion of proinflammatory cytokines, enhance release of pro-resolving mediators. The reprogramming of macrophages from classically (M1) to alternatively (M2) activated cells further amplifies the resolution cascade.]

Macrophages (MΦ) are heterogenous family of innate immune cells (Lavin & Merad, 2013). MΦs are tissue-resident or infiltrated/inflammatory hemopoietic cells of myeloid origin, known to exist in every tissue of the body, and exhibit organ specific functions (Lendeckel et al., 2022). Macrophages are phagocytic in nature, perform a diverse array of functions by integrating the cues from various cellular signals that arise in response to varied stimuli (injury/pathogen) (Mosser et al., 2021). Macrophages are essential for innate immunity, to maintain tissue integrity and homeostasis, in the development of normal tissue, and are also critical in resolution of inflammation (Italiani & Boraschi, 2015). They phagocytose apoptotic neutrophils by efferocytosis, help in repair of tissue following injury. They are also mediators in fibrosis, tumor growth and immunosuppression (Ge et al., 2022). MΦs exhibit their functions by promoting release of proinflammatory mediators, presenting antigens to naive T lymphocytes thereby stimulating adaptive immune response in the tissue (Di Benedetto et al., 2019). Macrophages are named based on their tissue precise location, such as Kupffer cells (liver), microglial cells (brain), Hofbauer cells (placenta), osteoclasts (bone), alveolar macrophages, pneumocytes type II (lung), Serous macrophages (serous cavities), histiocytes (connective tissue), Langerhans cells (LC) (skin), Synovial cells (type A) (Joints/cartilage) etc (Lendeckel et al., 2022). Additionally, macrophages can be classified into two varied phenotypes based on their functional heterogeneity as: classically stimulated pro-inflammatory macrophages (M1) and alternatively stimulated anti-inflammatory macrophages (M2) (Atri, et al., 2018). These phenotypic variants differ in their cytokine production, expression of receptors on their surface. Macrophages can be categorized or polarized to M1 pro-inflammatory phenotype by IFN-γ, tumor necrosis factor alpha (TNFα) and lipopolysaccharide (LPS) (Kosyreva et al., 2021). Activated M1 macrophages stimulate production and release of TNFα, L-6, IL-12, IL-1β and IL-23, along with monocyte chemotactic protein 1 (MCP-1), chemokine CCL8, macrophage inflammatory protein 2 (MIP-2), ROS, nitric oxide (NO), CD16, and CD32 (Kolliniati et al., 2022) as depicted in Figure 1. M1 polarization of macrophages is essential for viral clearance, enhance antiviral immunity due to release of cytokines and infiltration of polymorphonuclear leukocytes (PMN) and dendritic cells, have robust antimicrobial, antitumor activities, and mediate TH1 response. The polarization of M2 macrophages, occurs in response to transforming growth factor beta (TGF-β) and TH2 cytokines (IL-4, IL-13) and anti-inflammatory cytokines IL-10. M2 macrophages perform immune regulation, anti-inflammation, wound healing and tissue repair (Murray and Wynn, 2011). M2 macrophages exhibit enhanced levels of IL-1 receptor antagonist (IL-1RA), arginase 1 (Arg-1), IL-10, TGF-β, Chitinase-3-Like Protein 3 or Ym1,CCL18, and low production of IL-12 phenomenon. M2 macrophages also express CD206 (C-Type Mannose Receptor 1) and CD163 (Hemoglobin-Haptoglobin Scavenger Receptor) (Rőszer, 2015). The polarization to pro-inflammatory and antimicrobial M1 macrophages must be inhibited to prevent further collateral host tissue damage.

SARS-COV2 caused disastrous COVID-19 pandemic that claimed several millions of lives. The overwhelmed death rate associated with COVID-19 was mainly attributed to acute respiratory distress syndrome (ARDS), cytokine storm and fatal multi-organ failure. Other pathological events observed in COVID-19 cases are disseminated intravascular coagulation (DIC) syndrome, edema, and pneumonia, which are prime consequences of macrophage activation syndrome (MAS) (Hojyo et al., 2020). Angiotensin-converting enzyme 2 (ACE2) receptors are essential for entry of SARS-COV2, and are found to be prominently expressed on the surface of alveolar macrophages (AMs), suggesting that lungs are the prime direct target of SARS-CoV-2 mediated pathogenesis (Dockrell et al., 2022). Lung macrophages include two subtypes: alveolar macrophages which lie in close vicinity to type I and type II epithelial alveolar cells, and interstitial macrophages (IMs), which are located between alveolar epithelial zone and capillary bed (Shirey et al., 2014; Abassi et al., 2020). Ontogeny of both subtypes varies, IMs are derived from adult hematopoiesis and replenished from blood Ly6Chi (lymphocyte antigen 6c) monocytes. Moreover, a large proportion of AMs are derived from fetal liver monocytes, with smaller proportions from yolk sac–derived progenitors and bone marrow-derived monocytes (Guilliams & Svedberg, 2021). Nevertheless, in presence of an inflammatory stimuli AMs can be repopulated from circulating blood Ly6Chimonocytes. Additionally both IMs and AMs can be categorized in to two phenotypes (M1 and M2), therefore, M1 AMs, M2 AMs and M1 IMs and M2 IMs are different subtypes known to exist (Tamoutounour et al., 2013).

AMs are located in pulmonary airspaces, constitute nearly ∼95% of the alveolar immune cells. AMs are crucially required to maintain lung homeostasis *via* clearing pulmonary surfactant and cellular debris, essential for tissue repair and resolution of inflammation (Bissonnette et al., 2020). They phagocytose inhaled irritants and are the first cells to respond to pathogens, hence are known as alveolar guardians/scavengers. CD68 (“scavenger receptor”) was highly expressed in AMs. AMs phagocytose invading pathogen, entering lungs in lesser proportion, by crawling *via* pores of Kohn under influence of chemotaxis (Neupane et al., 2020). In physiological state, AMs exist in M2 phenotype articulating the cell-surface protein mannose receptor C type 1 (MRC1, CD206). Moreover, in pathogenic conditions i.e. in response to varied stimuli (injury/pathogen), AMs rapidly shift from M2 phenotype to M1 phenotype owing to their highly plastic nature. In SARS-COV2 viral RNA or DNA components act as pathogen connected molecular-patterns—(PAMPs), which activate AMs toward the M1 phenotype of macrophages readily (Wang et al., 2022). M1 AMs enable the SARS-CoV-2 to smoothly enter, and replicate in the lungs. This might be due to low endosomal pH and enhanced cellular softness. NP a structural protein of SARS-CoV-2 is essential for the assembly of the nucleocapsid and the release of virions, which is highly expressed in M1AMs (Lv et al., 2021). CD206 is a typical M2 marker limiting viral spread. Intriguingly, M2 AMs exist in lower lysosomal pH, mobilize more acidic lysosomes for viral degradation, thereby limit SARS –COV2 spread i.e.M2 AMs exhibit antiviral/anti-inflammatory effects (Lv et al., 2021; Zhang et al., 2021).

### SPMs in COVID-19

Infection with SARS-CoV2 is attributed with increase in chemical mediators such as IL-6, C-reactive protein, D-Dimers, fibrinogen and erythrocyte sedimentation rate (Huang et al., 2020). In COVID-19 infections, there is exaggerated production of proinflammatory lipid mediators like cytokines, chemokines and leukotrienes (LTB4) (Han et al., 2020; Huang et al., 2020), also it is prominently observed that endogenous SPM production is dysregulated *in vivo* (Palmas et al., 2021). It is reported that SARS-CoV-2 infected patients are at an increased risk due to thrombotic events. Hence management of COVID-19 patients with prophylactic anticoagulant or thrombolytic agents is crucial. It was reported that administration of resolvin D4 (RvD4), an SPM, stimulated thrombus resolution by decreasing neutrophil infiltration, and upregulating apoptosis of PMN, promoting further synthesis of D-series resolvins involved in inflammation resolution (Cherpokova et al., 2019). Resolvin D1 and Resolvin D2 are known to reduce viral spike protein induced production of pro-inflammatory cytokines and chemokines (Recchiuti et al., 2021). Lipoxin (LXA4), methyl ester-benzo-lipoxin A4 (BLXA4), resolvin (RvE1) may be used to treat gingival inflammation (Hasturk et al., 2021). In COVID-19 patients with cardiovascular complications, the omega-3 essential fatty acids can be used as an adjuvant therapy (Darwesh et al., 2021). Interestingly in a randomized clinical trial using high-dose n-3 PUFA supplementation (1.5 g/day EPA and 1.0 g/day DHA), it was reported that, n-3 PUFAs might exert direct modulatory effects on the chemical mediators responsible to cause the cytokine storm such as IL-6, IL-1β and TNF-α (Tan et al., 2018). Resolvins inhibit neutrophil infiltration by decreasing expression of surface adhesion receptors such as CD11b, CD18 on PMN, also capable of decreasing the release of IL-8chemokine.Resolvin E1 inhibits mitogen -activated protein kinase (MAPK)and NF-κB pathways, reduce PMN infiltration and cytokine release and can be used for treatment of sepsis induced cardiomyopathy (Zhang et al., 2020). Maresin1 and its epoxide intermediate 13S, 14S-epoxi-DHA, enhance the macrophage conversion from M1 to M2 phenotype, it is well known that M2 are pro-resolving in nature, enhance macrophage phagocytosis and efferocytosis (Freedman et al., 2020). Lipoxins (LXs) might reduce the inflammatory changes that occur due to activation of MAPK, mTOR, and NLPR3 inflammasome in COVID-19, reduce tumor necrosis factor-alpha and interferon-gamma secretion, block T cell migration and promote T cell apoptosis (Batiha et al., 2022). Neuroprotectin D1/Protectin reduce PMN infiltration through endothelial cells and stimulate the removal of apoptotic cells by MΦ t have crucial hereby play a crucial role in managing diseases such as Alzheimer’s disease (Rajasagi et al., 2013; Mohammed et al., 2022). It was reported that SPMs could enhance antiviral B lymphocytic activity during viral infections like influenza. Additionally, SPM precursors such as 17-hydroxy docosahexaenoic acid (17-HDHA) enhance immune responses against influenza infections hence could be used as vaccine adjuvants (Ramon et al., 2014).

These findings using several *invivo* and *invitro* models highlight the therapeutic potential of SPMs. They can be used as adjuvants to anti-inflammatory agents, or can act as agonists and enhance the pro-resolving actions of endogenous mediators. Hence can be best used as novel therapeutic molecules to combat infection as in the case of COVID-19.

### SPMs in macrophage regulation

Macrophages are the crucial players of the inflammatory milieu that arise due to COVID-19. They are regarded as the first line of immune defense, as they mediate between innate immunity and adaptive immunity responses. Moreno-Eutimio et al., demonstrated that host toll-like receptors (TLRs) specifically TLR7/8 is an invader sensing receptor able to recognize ssRNA sequences of SARS –COV2, this recognition could stimulate M1 pro-inflammatory macrophages which may trigger a rapid type I IFNs(interferon) response, consequently causing innate immune hyperactivation, aberrant cytokine production and ultimately lead to acute lung injury (ALI), ARDS, multi-organ fibrosis and dysfunction (Moreno-Eutimio, et al., 2020). Elevated levels of infiltrated macrophages with subsequent overproduction of pro-inflammatory cytokines are correlated with clinical manifestations of COVID-19.

Lipoxins (LXs), can inhibition of viral entry and its replication, downregulate ACE2 receptors, inhibit release of the pro-inflammatory cytokines. Moreover, LXs can augment the polarization of macrophages from (M1) to (M2) phenotype, causing a shift from pro-inflammatory to anti-inflammatory environment, thereby enhancing the inflammation resolving actions of macrophages in SARS-CoV-2 infection (Das, 2021; Batiha et al., 2022). RvE1and RvE4 enhances macrophage phagocytosis and efferocytosis of senescent erythrocytes that characterize COVID-19 (Libreros et al., 2021). RvE3 decreases allergic airway inflammation *via* the IL-23/IL-17A pathway (Sato et al., 2019).

During acute inflammation, biosynthesis of SPMs, specifically resolvin D1 [RvD1] and RvD5) was enhanced due to overexpression of miR-466l in macrophages, which ultimately enhanced resolution of inflammation, efficiently demonstrated in mice models by Li et al. (Li et al., 2013). RvD1 and RvD2 subside MΦ-driven inflammation *via* potentiating MΦ phagocytosis, decreasing release of pro-inflammatory cytokines and chemokines from MΦ, in response to the viral spike protein (Recchiuti et al., 2021). Maresins (MaRs) can resolve inflammation by stimulating macrophage phagocytosis of neutrophils (Weill et al., 2020). Maresins 13S, 14S-epoxy-maresin intermediate generated during synthesis of Maresin (MaR1) is bioactive and stimulates the phenotype transition from M1 to M2 macrophages (Serhan et al., 2022). RvD6 isomer and Elovanoid-N32 reduced expression of the ACE2 receptors and blocked the receptor binding domain of the virus spike protein thereby preventing receptor-virus interactions, reducing pro-inflammatory cytokine release (Pham et al., 2021). The protectin (PD) family of mediators regulate viral propagation by inhibiting intracellular viral RNA transport mechanisms. Neuroprotectin D1/Protectin D1 exerts anti-inflammatory/pro-resolving effects by suppressing oxidative stress, Aβ42 production as observed in pathologies like retinal degenerations, stroke, and Alzheimer’s disease (Bazan, 2009).

### SPMs in cytokine regulation

SARS-CoV-2 triggers cytokine production in overabundance, resulting in exaggerated inflammatory response and an avalanche of cytokines, dubbed “cytokine storm” (CS) or “quasi cytokine storm” in patients with COVID-19 (Gallo et al., 2022). CS can lead to persistent fever, muscle pain, hypotension, endothelial dysfunction, thromboembolic events, hypercoagulation, and cardiovascular complications, and in even more severe cases, to ALI/ARDS, hemophagocytic lymphohistiocytosis (HLH), multiple organ dysfunction and eventually death (Yang, L et al., 2021; Yang et al., 2020). CS can, therefore, serve as a measure of disease progression and severity (Tang Y et al., 2020). SARS-CoV-2 infection was found to result in elevated levels of pro inflammatory mediators such as interleukins (IL) (a pro-inflammatory cytokine mediator), tumour necrosis factor-α (TNF-α), CCL5, CXCL9, CXCL10, and CXCL11. Among IL, increase in levels were specifically found among IL-1β, IL-6, IL-7, IL-8, IL-9, IL-10, IL-13, IL-15, IL-17, IL-18, IL-29, IL-1ra, IL-2, IL-2Rα (Figure 1) (Huang et al., 2020; Jafarzadeh et al., 2020). Severe cases of the disease resulted in increased levels of CCL7, CCL3, and CXCL9, while quantities of CXCL10, CCL2, and CCl3 were higher in patients admitted to the ICU. Compared to this, moderate cases showed elevated levels of IL-18, IP-10, and M-CSF. IL-6, IL-7, IL-10, G-CSF, IP-10, MCP-1, MIP-1α, IL-18, MCP-3, M-CSF, and MIG. Other than these, CXCL12, CXCL1, CCL11, and the chemokine CCL27 were also elevated in COVID-19 patients (Chi et al., 2020). Another study reported higher serum levels of IL-6, IL-7, IL-10, granulocyte colony-stimulating factor (G-CSF), M-CSF, IP-10, monocyte chemoattractant protein-1 (MCP-1), MCP-3, MIG, and macrophage inflammatory protein 1α (MIP1α) is severe COVID-19 patients. All of these cytokines might be useful in predicting the severity of the disease (Catanzaro et al., 2020). Initial studies on COVID-19 patients showed that cytokine levels were enhanced in ICU patients as compared to non-ICU patients (Huang et al., 2020). In another study it was reported that sharply elevated IL-6 cytokine levels were observed in critically ill patients (Chen X et al., 2020; Angriman et al., 2021).

Pro-resolving mediators such as SPMs can be used to counter CS in cases of severe COVID-19 (Lee, 2021). Resolvin D1 and Resolvin D2 are especially useful in this regard due to their ability to reduce the levels of TNF-α, IL-8 and MIP-1, and attenuate the effects of CS (Recchiuti et al., 2021). Other SPMs that can reduce cytokine production include: RvD1, which achieves this result by downregulation of the NF-κB pathway (Molaei et al., 2021); RvE1, which reduces the levels of IL-8 and TNFα production; and LXs, which might inhibit the TLR-4/MyD88 axis activated during COVID-19 and reduce hyperinflammation and CS-induced effects (Tang Y et al., 2020). LXs in particular are also known to inhibit IL-6 and TNF-α production, while LXA4 inhibits iNOS, NF-kB, MAPK and COX-2 and enhance NRF2 genes expression (Zhang et al., 2022). All these SPMs could be of therapeutic application during SARS-COV-2 infection. Other SPMs with similar benefits include Resolvin E1 and LXA4 (capable of suppressing IL-23 and IL-17, IL-6 and TNF-α); PDX and NPD1 (decreases secretion of TNF-α, IL-6, IL-1β, CXCL-1, CXCL-2 and NOS-2) (Bosviel et al., 2017). Finally, there are RvD2 and Mar1, called block inflammasome components, and can cause notable reduction in potent pro-inflammatory cytokine IL-1β (Lopategi et al., 2019).

## Conclusion and future prospective

The major reason for severity of COVID-19 infection is attributed to the acute uncontrolled inflammatory response in the lungs known as cytokine storm. The macrophages play important role in regulating COVID-19 inflammation by switching between two forms: pro-inflammatory macrophages (M1) and anti-inflammatory macrophages (M2) forms. Since the lungs are preferred target of most respiratory viruses including COVID-19 so the involvement of lung alveolar macrophages in progression of severe SARS-COV 2 is eminent. ACE2 receptors are essential for entry of SARS-COV2, and are found to be expressed on alveolar macrophages (AMs). The shifting of alveolar macrophages from M2 to M1 form triggers the SARS-COV 2 virus to smoothly infect the lungs, replicate and consequently induce cytokine storm. This causes build-up of oedema i.e. accumulation of inflammatory fluids in lungs that averts oxygen transfer from lungs towards blood capillaries inducing hypoxia and if untreated then associated multiple organ failure. The specialized pro-resolving lipid mediators are found to be highly effective in inhibiting inflammatory responses during infection. They are produced by innate immune cells from essential long chain fatty acids and are found to improve the concentration of anti-inflammatory lipid mediators. The specialized pro resolving lipid mediators such as protectins, lipoxins and resolvins have been strongly associated with inhibition of cytokine avalanche or cytokine storm which is a well-known reason behind COVID-19 deaths. The viral diseases have been always difficult to treat due to numerous factors such as limited anti-viral pharmaceutical agents, frequent re-infections, frequent mutations in RNA viruses and restructuring of genome etc. but SPM’s offers a different approach of treatment wherein we can control severity of infection by controlling inflammation. This is well justified by the fact that most viral infections subside on their own after completion of their cycles in host. Infact the majority of Covid patients developed mild symptoms possibly due to absence of inflammatory responses as cytokine storm. So in near future regulating the respiratory inflammation shall be the goal in order to avoid progression to severe respiratory distress. Thus there is great scope of using SPM’s against COVID-19 variants and also other viral infections in near future particularly RNA viruses which evades novel pharmaceutical agents due to their mutagenic capabilities.
